# Anti-influenza A virus restriction factors that shape the human species barrier and virus evolution

**DOI:** 10.1371/journal.ppat.1011450

**Published:** 2023-07-06

**Authors:** Philipp Peter Petric, Martin Schwemmle, Laura Graf

**Affiliations:** 1 Institute of Virology, Medical Center–University of Freiburg, Freiburg, Germany; 2 Faculty of Medicine, University of Freiburg, Freiburg, Germany; University of Wisconsin-Madison, UNITED STATES

## Introduction

Zoonotic infections with influenza A viruses (IAVs) of avian origin can cause severe disease with high fatality in humans, as in the case of the avian IAV subtypes H5N1 and H7N9. Fortunately, such spillover events from the avian reservoir to humans are rare and mostly limited to single individuals due to a robust species barrier. The molecular basis for this species barrier is the intrinsically poor adaptation of avian IAVs to humans [1]. Avian IAVs are unable to exploit human host factors required for virus replication and to escape anti-IAV restriction factors that serve as gatekeepers for zoonotic IAVs in human cells. As a result, efficient virus replication is hampered and virus spread within the human population is not readily possible. IAVs, however, constantly evolve. Occasionally, they can acquire the capacity to overcome the human species barrier. This is a very rare event. Yet, all past and current seasonal IAVs circulating in the human population can be traced back to animal reservoirs. IAVs possess an error-prone polymerase that facilitates the acquisition of mutations in the viral genome. Most of these mutations are detrimental for the virus, but some may favor interactions with pro-viral host factors or bypass specific antiviral factors and may be selected because they grant a fitness advantage in the new host [2]. Numerous adaptation steps are required to overcome the species barrier and to achieve sustained circulation in the human population. For zoonotic IAVs of avian origin, the step-wise acquisition of favorable mutations is a major challenge. However, preadaptation in intermediate hosts, such as pigs may facilitate the adaptive process. Another possibility for avian IAVs to overcome the human species barrier is the exchange of viral genome segments with viral strains already adapted to humans by reassortment that may occur in coinfected cells. Examples include the pandemic viruses that caused the Asian influenza of 1957 or the Hong-Kong influenza of 1968. Pigs may play a special role in reassortment because they are equally susceptible to infections with avian and human IAVs and thus provide an ideal environment for the emergence of new strains with pandemic potential. Indeed, multiple reassortments between human, porcine, and avian IAVs over several years led to the 2009 IAV pandemic [2,3].

Many different host factors that are essential for efficient viral replication of IAV in human cells have been described to also contribute to the human species barrier preventing zoonotic IAV infections (for a review see [4]). Here, we review current insights into human, anti-IAV restriction factors that preferentially inhibit zoonotic IAVs and discuss how preadaptation in intermediate hosts may enable IAVs to escape them. In addition, we address how genetic defects in the antiviral response can compromise the human species barrier.

### Which anti-IAV restriction factors do preferentially block IAVs originating from nonhuman species?

Most known anti-IAV restriction factors do not differentiate between IAVs of human and animal origin [5]. A notable exception is the interferon (IFN)-induced human myxovirus resistance protein A (MxA or hsMx1), a dynamin-like large GTPase that interferes with viral genome replication (for a review see [6]). MxA recognizes the viral nucleoprotein (NP) and blocks the import of viral ribonucleoprotein complexes (vRNPs) and newly synthesized NP into the nucleus of infected cells [7,8]. Experiments in cell culture and in MxA-transgenic mice revealed that pandemic as well as seasonal human IAV strains are less sensitive to MxA-mediated restriction than strains of avian, swine, bat, and equine origin. This MxA-escape phenotype was assigned to the viral NP [9–14]. Likewise, Mx1 proteins are part of the species barrier against IAVs in other mammalian species. Equine Mx1 blocks avian and human IAVs, whereas equine IAVs have escaped from restriction [12]. Similarly, porcine Mx1 inhibits avian IAVs, but is only weakly active against swine or human IAVs [11,13]. These observations highlight a major role of Mx1 proteins in preventing inter-species transmissions of IAVs. Another IFN-regulated restriction factor with selective antiviral activity against avian strains in humans is butyrophilin subfamily 3 member A3 (BTN3A3). It blocks viral transcription of avian but not human IAVs and similar to Mx proteins, NP was identified as the determinant of the high sensitivity of avian IAVs to BTN3A3 [15]. Interestingly, BTN3A3 orthologs of other species, including pigs, ducks, and chickens, show no antiviral activity against IAVs of avian or human origin [15]. Moreover, a CRISPR activation screen in human cells recently identified the glycosyltransferase B4GALNT2 as a cellular factor that specifically blocks viral entry of avian IAVs [16]. B4GALNT2 modifies glycans terminating in α2-3-linked sialic acid, the receptor of avian IAVs, thereby preventing entry of avian IAVs. Human IAVs, however, circumvent this antiviral strategy due to their α2-6-linked sialic acid receptor specificity. Finally, the human splicing regulator TRA2A has been shown to restrict avian IAVs while it promotes replication of human IAVs by affecting the splicing of the M and NS segments [17]. TRA2A binds to an intronic splicing silencer (ISS) in the M mRNA of avian IAVs thereby inhibiting viral M2 expression and progeny production. Conversely, human IAVs carry an ISS in the NS mRNA. Impeded NS mRNA splicing is required for a balanced expression ratio of the viral IFN antagonist NS1 and the nuclear export factor NEP leading to optimized viral gene expression and replication [17]. This study supports previous findings showing how important balanced splicing of the NS mRNA is for efficient replication of zoonotic IAVs in the human host [18]. Together, these examples illustrate how diverse the inhibitory mechanisms of human restriction factors are that specifically prevent human infections by avian IAVs.

### How do zoonotic IAVs evade human restriction factors?

**Fig 1:**
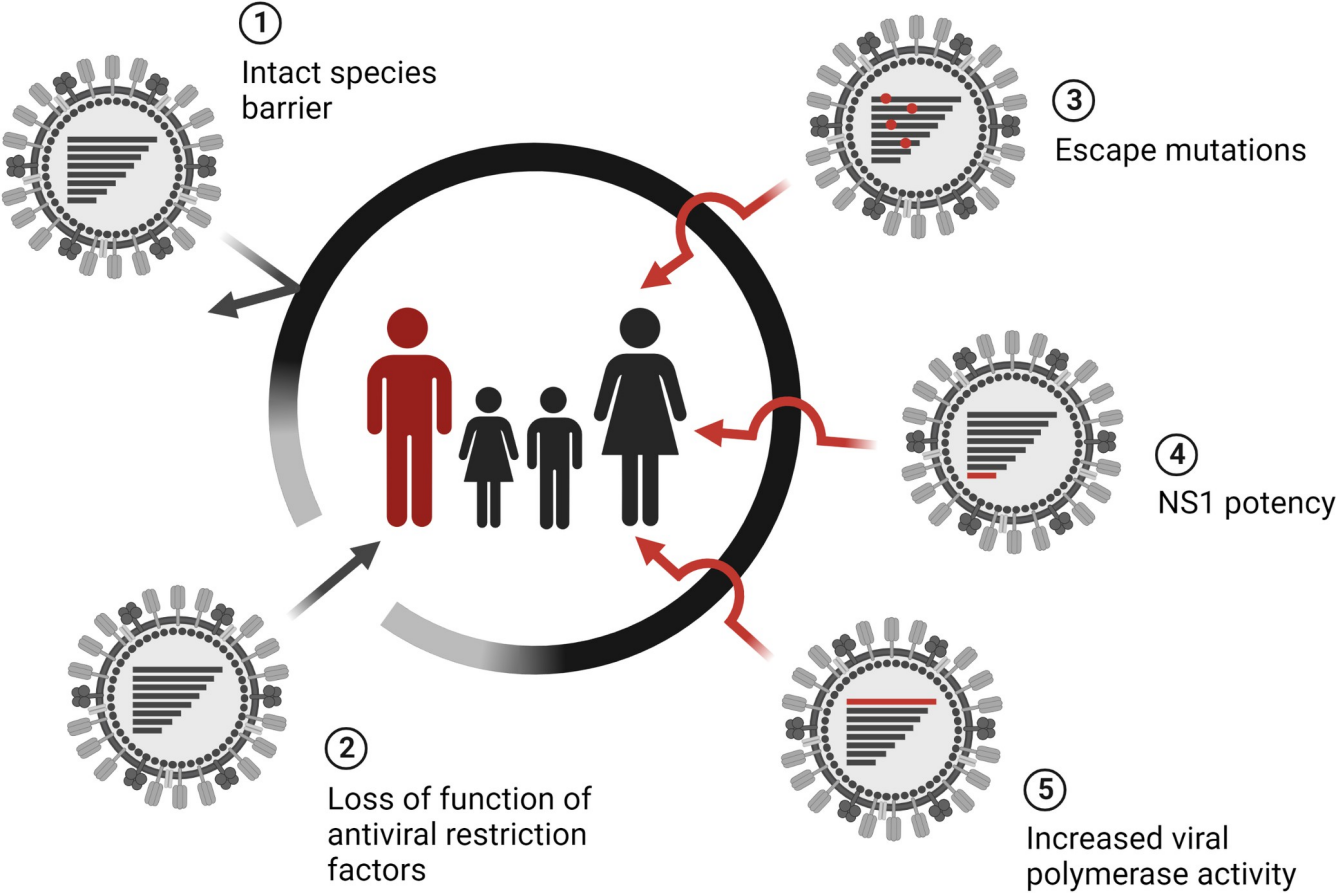
How zoonotic IAVs can overcome anti-IAV restriction factors. Avian IAVs are poorly adapted to the human host. This species barrier prevents individual infections (1). Deficiencies of anti-IAV restriction factors or defects in their activation like loss of IFN induction or signaling required for the expression of antiviral ISGs can increase the susceptibility of individual humans to zoonotic IAVs (2). Zoonotic IAVs can also exploit different adaptation strategies to evade antiviral restriction factors including the acquisition of escape mutations (3) or replication enhancing mutations resulting in highly potent NS1 variants antagonizing the IFN response (4) or increased viral polymerase activity (5). Created with BioRender.com. IAV, influenza A virus; IFN, interferon; ISG, IFN-stimulated gene.

Escape from restriction factors of the species barrier is a prerequisite for zoonotic IAVs to establish a new lineage in the human population (Fig 1). Thus, a common characteristic of all human IAVs is their drastically reduced sensitivity to MxA restriction due to adaptive mutations in NP, the viral target of MxA [10,13]. The combinations of amino acid substitutions mediating MxA escape vary between different IAV lineages. While the main escape mutations in NP of the 1918 Spanish H1N1 virus (100I/V, 283P, and 313Y) and the 2009 pandemic H1N1 strain (53D, 100I/V, and 313V) and their seasonal descendants are very similar, the MxA escape mutations for the Eurasian avian-like swine influenza A (EA) lineage differ greatly (48Q, 98K, and 99K) [11,13]. An unbiased deep mutational scan confirmed these substitutions and identified additional NP mutations conferring MxA escape that have not been detected in virus isolates so far [19], highlighting the plasticity of MxA escape signatures in NP. However, acquisition of MxA escape mutations causes severe virus attenuation due to impaired nuclear import of vRNPs and requires additional stabilizing mutations to compensate for this fitness loss [13,20]. This observation emphasizes the high hurdle of the species barrier due to MxA that can only be overcome by a multistep mutational process. Interestingly, escape from BTN3A3 requires NP substitutions identical to those previously associated with MxA escape (313Y/V and 52N/H/Q) [13,15,21]. Unlike MxA, only 1 adaptive mutation is required to escape from BTN3A3. Since acquisition of this adaptive mutation does not affect viral fitness, zoonotic IAVs may rapidly overcome this species barrier [15]. Despite targeting the same surface exposed area of NP, BTN3A3 is antivirally active independently of MxA [15]. Adaptation of avian IAVs to mammalian hosts also requires redirecting the binding of the splicing inhibitor TRA2A from viral M mRNA to NS mRNA. Adaptive mutations in the ISS of the viral M gene have been observed, for example, in human isolates of the zoonotic avian IAV H5N1 [17].

Partial escape from anti-IAV restriction factors can also be achieved by genetic changes that enhance viral fitness overall which, in turn, increases replication in the human host and may facilitate the acquisition of additional, true escape mutations (Fig 1). The virulence factor NS1 of IAV leads to diminished expression of IFN-stimulated genes (ISGs), including MxA and BTN3A3. NS1 variants with enhanced IFN-antagonistic properties have been detected in avian-origin H5N1 viruses isolated from birds and humans [22]. Furthermore, mutations that increase viral polymerase activity can result in enhanced viral replication capable of overrunning the host innate immune response [23]. When an MxA-sensitive, avian-origin H7N9 patient isolate was experimentally adapted to grow in immunocompromised mice that express a human MxA transgene, it did not acquire MxA escape mutations in NP. Instead, it acquired a mammalian adaptive mutation in the viral polymerase subunit PB2 (E627K) [24]. Although this mutation did not confer MxA escape, it enabled increased viral replication in MxA-transgenic mice.

### What is the role of intermediate hosts in adaptation of IAVs to human MxA?

Acquiring the necessary set of MxA escape mutations appears to be particularly challenging because the concomitant fitness loss needs to be compensated. Consequently, without the strong selective pressure of MxA, the MxA escape phenotype will gradually be lost. The 2009 pandemic H1N1 strain, for example, lost some of the MxA escape-mediating mutations in NP after reintroduction into the swine population due to the reduced antiviral pressure by porcine Mx1 [11,13]. On the other hand, the comparatively weaker antiviral activity of Mx1 proteins in intermediate IAV hosts can promote gradual preadaptation to human MxA. This has been observed for the precursor of the 2009 pandemic H1N1 lineage and the EA lineage in pigs [11,13]. Preadaptation to porcine Mx1 in pigs lowered the species barrier for the pandemic H1N1 precursor virus by acquiring partial MxA escape while the full MxA escape genotype was obtained in the human population [13]. The avian Mx1 proteins analyzed so far in duck and chicken show no anti-IAV activity [25,26]. However, the relatively low sensitivity of avian IAVs to porcine Mx1 may explain why pigs appear to be an ideal host, facilitating the acquisition of intermediate levels of Mx-resistance for otherwise MxA-sensitive IAVs. Similarly, a recently identified IAV from bats, H18N11, shows some escape from MxA that may originate from preadaptation during replication in the presence of bat Mx1 [9,27]. In contrast, in horses, preadaptation of equine IAVs to human MxA has not been observed so far. While equine IAV strains show strong resistance to equine Mx1, they are still sensitive to the antiviral activity of human MxA [12].

Another way for IAVs to gain MxA escape is their reassortment with MxA-adapted IAVs in intermediate hosts, such as pigs. Interestingly, the majority of reassortants that circulate in pigs carry NP segments with MxA escape mutations. These genomic segments were derived from 2009 pandemic H1N1 viruses that were accidentally reintroduced into pig herds [28]. The pandemic H2N2 of 1957 and the pandemic H3N2 virus of 1968 both escaped the MxA barrier by acquiring the already human-adapted NP genomic segment from the descendant of the 1918 pandemic H1N1 virus. It is still unclear in which hosts the reassortments took place [29] and how MxA escape arose in the 1918 virus strain. A recent study analyzing archival 1918 H1N1 genomes from formalin-fixed lungs suggests that the complete set of MxA escape mutations in NP was acquired in humans during the ongoing pandemic. In early isolates only 100I and 313Y were detected, whereas later isolates additionally gained 283P as well as the stabilizing amino acid 16D [30].

### Do anti-IAV restriction factors play a role in the susceptibility of humans to avian IAVs?

Among the best-studied anti-IAV restriction factors associated with IAV susceptibility is the IFN-induced transmembrane protein 3 (IFITM3), an entry inhibitor of avian and human IAVs. Genetic variations within the *IFITM3* gene have been shown to be associated with the severity of seasonal and pandemic IAV infections [31–33]. In addition, in patients infected with the avian IAV subtype H7N9, the homozygous C/C genotype of the single-nucleotide variant (SNV) rs12252 was associated with an increased risk for a severe clinical outcome and death [34,35]. Thus, IFITM3 appears to be a critical restriction factor reducing disease severity in patients infected with either human or avian IAVs, but does not seem to play an essential role for the species barrier, in contrast to MxA. A recent study demonstrated that MxA reduces the risk of infections with avian IAVs in humans. By comparing whole-genome sequences of patients infected with the avian IAV subtype H7N9 with those of healthy poultry workers, Chen and colleagues observed a strong association between H7N9 infection and rare, heterozygous SNVs in the MxA-encoding *MX1* gene [36]. About 6.5% of the H7N9 patients, but none of the healthy controls, were carriers of antivirally inactive MxA variants that exhibited a dominant-negative phenotype and interfered with the function of wild-type MxA. Hence, heterozygous carriers had an MxA-null phenotype and an inherently increased risk for avian IAV infections. The underlying cause for increased IAV susceptibility in the remaining patients who had a wild-type *MX1* gene is unclear. One explanation might be defects in IFN induction and signaling hampering the expression of MxA and BTN3A3 (Fig 1). Inborn errors of the IFN system have been previously detected in patients with life-threatening, seasonal influenza [37]. The identification of individuals with increased susceptibility to zoonotic IAVs due to genetic defects is demanding since it depends on how rare these defects are and whether the patient cohort is large enough although IAV zoonoses are sporadic.

## Conclusions

Anti-IAV restriction factors such as the MxA protein are major components of the human species barrier against transmission of zoonotic IAVs. Most likely, numerous additional host restriction factors are involved and need to be discovered. Such antiviral barriers can be overcome through the acquisition of escape mutations in the viral genome, host genetic defects that compromise innate antiviral defenses, and reassortment events. Preadaptation, including reassortment, in intermediate hosts can also increase the viral fitness of nonhuman IAVs, thereby increasing their zoonotic and pandemic potential. It will be important to monitor the capacity of IAVs originating from diverse animal reservoirs to escape human restriction factors. It will be equally important to identify and protect highly susceptible individuals carrying inactive variants of these essential antiviral host factors.
